# Excesso de mortalidade geral e mortalidade por COVID-19 no Brasil e regiões no ano de 2020

**DOI:** 10.1590/0102-311XPT217323

**Published:** 2025-01-13

**Authors:** Laylla Ribeiro Macedo, Carolina Borges de Araújo, Luciana Freire de Carvalho, Jackeline Christiane Pinto Lobato, Natália Santana Paiva, Antonio Joseé Leal Costa

**Affiliations:** 1 Instituto de Estudos em Saúde Coletiva, Universidade Federal do Rio de Janeiro, Rio de Janeiro, Brasil.; 2 Instituto de Saúde Coletiva, Universidade Federal Fluminense, Niterói, Brasil.

**Keywords:** COVID-19, Excesso de Mortalidade, Estudos Ecológicos, COVID-19, Excess Mortality, Ecological Studies, COVID-19, Mortalidad Excesiva, Estudios Ecológicos

## Abstract

O objetivo deste estudo foi avaliar o excesso de mortalidade geral e a mortalidade por COVID-19 nas regiões do Brasil, no ano de 2020, segundo sexo e faixa etária. Realizou-se estudo ecológico que calculou o excesso de mortalidade geral, por sexo e faixa etária, utilizando os quantitativos esperados de óbitos em um contexto sem a pandemia e os óbitos observados em 2020. Dados sobre óbitos foram extraídos do Sistema de Informações sobre Mortalidade e dados populacionais do Instituto Brasileiro de Geografia e Estatística. O excesso de mortalidade foi calculado considerando a diferença entre as taxas de mortalidade observadas em 2020 e a média das taxas de 2015 a 2019, padronizadas por idade, e a diferença entre os óbitos observados obtido por meio de uma modelagem *quasi*-Poisson e aqueles esperados para 2020. No Brasil, a taxa de mortalidade geral padronizada, no ano de 2020, foi de 590 óbitos por 100 mil habitantes, sendo o excesso de mortalidade de 44 óbitos por 100 mil habitantes, enquanto a taxa de mortalidade por COVID-19 foi de 79 óbitos por 100 mil habitantes. As maiores taxas de mortalidade geral foram observadas nas regiões Norte e Nordeste. O excesso de óbitos estimado pela razão entre os óbitos observados e esperados, em todo o país em 2020, foi de 16%; sendo 17% para o sexo masculino, 16% para o feminino, 7% para indivíduos entre 0 e 59 anos, e 20% para aqueles com idade igual ou superior a 60 anos. Os dados permitiram um entendimento mais direcionado do impacto da pandemia de COVID-19 na mortalidade do Brasil em 2020, apontando um excesso de óbitos mais expressivo nas regiões Norte, Nordeste e Centro-oeste e entre os grupos de homens e com idade superior a 60 anos.

## Abstract (en)

This study aimed to evaluate the overall excess mortality and COVID-19 mortality in the regions of Brazil, in 2020, by sex and age group. An ecological study was carried out to calculate the overall excess mortality, by sex and age group, using the expected number of deaths in a non-pandemic context and the deaths observed in 2020. Data on deaths were extracted from the Brazilian Mortality Information System, in addition to population data from the Brazilian Institute of Geography and Statistics. Excess mortality was calculated considering: the difference between the mortality rates observed in 2020 and the average of the rates from 2015 to 2019, standardized by age; and the difference between observed deaths obtained via a quasi-Poisson model and the deaths expected for 2020. In Brazil, the standardized overall mortality rate in 2020 was 590 deaths per 100,000 inhabitants, with excess mortality of 44 deaths per 100,000 inhabitants, while the mortality rate from COVID-19 was 79 deaths per 100,000 inhabitants. The highest overall mortality rates were observed in the North and Northeast regions. The excess deaths estimated by the ratio between observed and expected deaths nationwide in 2020 was 16%; of which 17% were males, 16% were females, 7% were individuals from 0 to 59 years old, and 20% were individuals aged 60 years or older. These outcomes enabled a better understanding on the impact of the COVID-19 pandemic on the mortality in Brazil in 2020, indicating a more pronounced excess mortality in the North, Northeast and Central-West regions and among men older than 60 years.

## Abstract (es)

El objetivo fue evaluar el exceso de mortalidad general y la mortalidad por COVID-19 en las regiones de Brasil, en el año 2020, según el sexo y el rango de edad. Se realizó un estudio ecológico que calculó el exceso de mortalidad general, por sexo y rango de edad, utilizando las cifras de muertes esperadas en un contexto sin pandemia y las muertes observadas en el 2020. Los datos sobre defunciones se extrajeron del Sistema de Informaciones sobre la Mortalidad brasileño y de los datos poblacionales del Instituto Brasileño de Geografía y Estadística. El exceso de mortalidad se calculó teniendo en cuenta: la diferencia entre las tasas de mortalidad observadas en el 2020 y las tasas promedio del 2015 al 2019, estandarizadas por edad; y la diferencia entre las muertes observadas obtenidas mediante modelos cuasi-Poisson y las esperadas para el 2020. En Brasil, la tasa de mortalidad general estandarizada en el 2020 fue de 590 muertes por 100.000 habitantes, con un exceso de mortalidad de 44 muertes por 100.000 habitantes, mientras que la tasa de mortalidad por COVID-19 fue de 79 muertes por 100.000. Las tasas de mortalidad general más altas se observaron en las regiones Norte y Nordeste. El exceso de muertes estimado por la relación entre muertes observadas y esperadas, en todo el país en el 2020, fue del 16%; 17% para hombres, 16% para mujeres, 7% para personas entre 0 y 59 años, y 20% para personas de 60 años o más. Los datos permitieron comprender mejor el impacto de la pandemia de COVID-19 en la mortalidad en Brasil en el 2020, señalando un exceso más significativo de muertes en las regiones Norte, Nordeste y Centro-Oeste y entre grupos de hombres y personas mayores de 60 años.

## Introdução

A emergência da COVID-19 gerou um impacto expressivo nas diversas áreas em todo o mundo ^1^. Para além das implicações na esfera da saúde e na morbimortalidade da população, a pandemia amplificou as desigualdades sociais e suscitou desafios nos âmbitos político, econômico e ambiental ^2^. Segundo a Organização Mundial da Saúde (OMS), até novembro de 2023, foram registrados aproximadamente 772 milhões de casos da COVID-19 no mundo, sendo mais de 37 milhões de notificações da doença acumuladas no Brasil ^3^.

De acordo com o estudo realizado com dados de 74 países e 266 localidades subnacionais, foram registrados 5,94 milhões de óbitos por COVID-19 entre 1º de janeiro de 2020 e 31 de dezembro de 2021; porém, os achados indicaram que a magnitude da pandemia foi três vezes maior que os óbitos notificados no período (cerca de 18 milhões de mortes) ^4^. O estudo ainda mostrou que a taxa global de mortalidade em excesso para todas as idades, nesse período, foi de 120,3 óbitos por 100 mil habitantes, sendo essa taxa superior a 300 óbitos por 100 mil habitantes em 21 países, localizados principalmente nas regiões do sul da Ásia, norte da África, Oriente Médio e Europa Oriental ^4^. No Brasil, os dados indicaram um excesso de aproximadamente 190 mil óbitos durante o ano de 2020 ^5^. Há uma lacuna no que se refere à apresentação desse indicador, considerando os subgrupos sociodemográficos e regionais.

A taxa de mortalidade específica por COVID-19 é um importante indicador para avaliar o impacto da pandemia nas estatísticas vitais; no entanto, alguns países enfrentam barreiras na contabilização e/ou no acesso a esses dados ^6^. No Brasil, o monitoramento dos indicadores epidemiológicos de doenças, incluindo a COVID-19, compreende uma das atribuições da vigilância em saúde ^7^; porém, o cenário vivenciado no país dificultou essa mensuração, tendo em vista a escassez de testes de identificação do SARS-CoV-2, especialmente no ano de 2020 ^8^ ^,^ ^9^. A estratégia nacional para o enfrentamento da doença, planejada e executada de forma incipiente, ocasionou a fragmentação do financiamento para aquisição e distribuição desses insumos ^9^, causando uma potencial subnotificação dos casos e óbitos por COVID-19 ^8^ ^,^ ^10^.

Diante disso, a OMS recomenda a utilização do cálculo do excesso de óbitos, caracterizado quando o grau de mortalidade mensurado na atualidade supera os níveis historicamente estabelecidos ^6^. Sendo assim, em comparação com o uso de mortes confirmadas por COVID-19, o excesso de mortalidade geral fornece uma representação mais fidedigna do cenário vivenciado, já que possibilita gerar as estimativas de mortalidade com mais precisão e, dessa forma, auxiliar no conhecimento da real magnitude do impacto da pandemia. Esse parâmetro, que permite estimar o efeito direto e indireto na mortalidade, possibilita ainda sua comparação entre as localidades e os subgrupos da população, e pode ser útil para auxiliar gestores no planejamento e acompanhamento das ações de controle diante desse panorama ^6^.

Logo, considerando a demanda pelo conhecimento do impacto concreto da pandemia de COVID-19, de forma direta e indireta, qualificando subgrupos demográficos e regionais, além da necessidade de fortalecimento dos sistemas de registros nacionais, este estudo teve como objetivo avaliar o excesso de mortalidade geral e a mortalidade por COVID-19 nas regiões do Brasil, no ano de 2020, segundo sexo e faixa etária.

## Métodos

Trata-se de um estudo ecológico descritivo com dados de mortalidade geral e por COVID-19 no Brasil em 2020, considerando o sexo e a faixa etária.

Os dados sobre os óbitos foram extraídos do Sistema de Informações sobre Mortalidade (SIM) ^11^, sendo incluídos na análise os óbitos por todas as causas, naturais ou externas; e excluídos os óbitos fetais, ocorridos no período estudado. As seguintes variáveis da Declaração de Óbito (DO) foram consideradas para análise: município de residência, data do óbito, sexo, idade e causa básica da morte.

O estudo estimou o excesso de mortalidade a partir do quantitativo esperado de óbitos em um contexto sem a pandemia (2015 a 2019) e o quantitativo observado de óbitos em 2020. O ano de 2020 foi considerado para essa análise por ser anterior ao início da vacinação no país.

De acordo com a OMS, o excesso de mortalidade representa uma situação em que o número de mortes está situado acima do esperado, segundo o padrão de mortalidade previamente observado na população ^6^. É calculado como a diferença entre o número de mortes que ocorreram e o número que seria esperado na ausência da pandemia com base em dados anteriores ^12^.

O excesso de mortalidade para o ano de 2020 foi calculado considerando dois métodos, estimados por meio: (1) da diferença entre as taxas de mortalidade observadas em 2020 e a média das taxas de 2015 a 2019; e (2) da diferença entre o quantitativo de óbitos observados e aqueles esperados para 2020, obtido por meio de uma modelagem *quasi*-Poisson. A escolha de cinco anos para a estimativa da mortalidade esperada foi baseada em práticas de vigilância bem estabelecidas ^13^.

No primeiro método (1), foram calculadas as taxas de mortalidade padronizadas para o ano de 2020 e a taxa de mortalidade média padronizada para o período de 2015 a 2019, obtida pela média das taxas padronizadas de cada ano. As taxas de mortalidade padronizadas também foram calculadas estratificadas por sexo (masculino e feminino) e por faixa etária (0 a 59 anos e 60 anos ou mais), considerando os óbitos nesses grupos e as populações de cada estrato. O excesso de óbitos para o Brasil e cada região foi obtido pela diferença entre a taxa de mortalidade padronizada no ano de 2020 e a taxa de mortalidade média padronizada para o período de 2015 a 2019, considerada como a linha de base, para o total e especificamente por sexo e faixa etária. Para a padronização das taxas, pelo método direto, foi considerada a população do Brasil em 2010, a partir dos dados do *Censo Demográfico* desse ano ^14^. Os dados demográficos de 2015 a 2020, utilizados para o cálculo das taxas, foram extraídos da projeção populacional do Instituto Brasileiro de Geografia e Estatística (IBGE) ^15^.

No método que utiliza a modelagem (2), a linha de base foi estimada a partir de dados históricos considerando as tendências anteriores, representando o número esperado de mortes em 2020. As estimativas do número esperado de óbitos em 2020 e seu respectivo intervalo de 95% de confiança (IC95%), para o Brasil e cada uma das regiões, foram obtidas por meio do uso de modelos aditivos generalizados *quasi*-Poisson, com o intuito de acomodar a sobredispersão dos dados de óbitos entre 2015 e 2019. Com o objetivo de capturar possíveis tendências não lineares na contagem dos óbitos ao longo do período estudado, a variável ano de ocorrência do óbito foi ajustada de forma não paramétrica (*spline*). A partir da modelagem descrita, estimou-se o número de óbitos esperados em 2020, em um contexto sem pandemia de COVID-19 para o Brasil e regiões, considerando o sexo e a faixa etária. Em seguida, foram estimados a razão entre o número de óbitos observados e esperados para 2020 e o excesso de óbitos com seus respectivos IC95% calculados a partir do emprego da técnica de *Bootstrap* ^16^.

Também foram apresentados os óbitos atribuídos à COVID-19 para o Brasil e regiões, segundo estrato de sexo e faixa etária, bem como calculada a taxa de mortalidade pela doença, padronizada pelo método direto, considerando a população do Brasil em 2010. Para a definição de óbito por COVID-19, foram considerados aqueles que apresentaram como causa básica no SIM a subcategoria B 34.2 da 10ª revisão da Classificação Internacional de Doenças (CID-10). Todas as análises foram realizadas utilizando o software RStudio, versão 1.4.1717 (https://rstudio.com/).

Considerando que o estudo foi realizado com dados de domínio público e sem identificação dos indivíduos, preservando a confidencialidade, não foi submetido à aprovação de um Comitê de Ética em Pesquisa, de acordo com a *Resolução nº 510/2016*, do Conselho Nacional de Saúde.

## Resultados

No Brasil, a taxa de mortalidade geral padronizada no ano de 2020 foi de 590 óbitos por 100 mil habitantes, sendo o excesso de mortalidade (a diferença entre as taxas) de 44 óbitos por 100 mil habitantes. Já a taxa de mortalidade por COVID-19 foi de 79 óbitos por 100 mil habitantes. As regiões Norte, Nordeste e Centro-oeste apresentaram taxas de mortalidade geral superiores à observada no país, respectivamente 696, 631 e 611 óbitos por 100 mil habitantes. As maiores diferenças entre as taxas observadas e esperadas, em 2020, foram verificadas nas regiões Norte (130 óbitos por 100 mil habitantes) e Nordeste (66 óbitos por 100 mil habitantes). As regiões Sudeste (37 óbitos por 100 mil habitantes) e Sul (-3 óbitos por 100 mil habitantes) mostraram uma diferença entre as taxas inferiores ao observado no Brasil. A maior taxa de mortalidade por COVID-19 no ano de 2020 foi observada na Região Norte (130 óbitos por 100 mil habitantes), enquanto a menor na Região Sul (53 óbitos por 100 mil habitantes) (Tabela 1).

**Tabela 1:** Taxa de mortalidade anual média padronizada de 2015 a 2019, taxa de mortalidade acumulada padronizada em 2020, diferença entre as taxas (excesso de mortalidade) e taxa de mortalidade padronizada por COVID-19 em 2020, no Brasil e regiões.

	Taxa de mortalidade média padronizada (2015-2019)	Taxa de mortalidade padronizada (2020)	Diferença entre as taxas (2020 *vs*. 2015-2019)	Taxa de mortalidade por COVID-19 (2020)
Brasil	546	590	44	79
Norte	566	696	130	130
Nordeste	565	631	66	82
Sudeste	535	572	37	77
Sul	528	525	-3	53
Centro-oeste	549	611	62	97

Nota: as taxas são expressas por 100 mil habitantes. Padronização das taxas pelo método direto, utilizando como padrão a população do Brasil em 2010.

No ano de 2020, a taxa de mortalidade padronizada no Brasil para o sexo masculino foi de 761 óbitos por 100 mil homens, enquanto para o sexo feminino foi de 446 óbitos por 100 mil mulheres. O excesso de mortalidade e a taxa de mortalidade por COVID-19 também foram superiores no sexo masculino comparadas ao sexo feminino. Para todas as regiões do país, observou-se o mesmo cenário descrito anteriormente. O menor excesso de óbitos foi observado na Região Sul (5 óbitos para o sexo masculino e -8 óbitos para o sexo feminino, por 100 mil homens e mulheres, respectivamente), e o maior na Região Norte (178 óbitos para o sexo masculino e 89 óbitos para o sexo feminino, por 100 mil homens e mulheres, respectivamente), para ambos os sexos (Tabela 2).

**Tabela 2:** Taxa de mortalidade anual média padronizada de 2015 a 2019, taxa de mortalidade acumulada padronizada em 2020, diferença entre as taxas (excesso de mortalidade) e taxa de mortalidade padronizada por COVID-19 em 2020, no Brasil e regiões, segundo sexo e faixa etária.

	Taxa de mortalidade média padronizada (2015-2019)		Taxa de mortalidade padronizada (2020)		Diferença entre as taxas (2020 *vs*. 2015-2019)		Taxa de mortalidade por COVID-19 (2020)	
	Masculino	Feminino	Masculino	Feminino	Masculino	Feminino	Masculino	Feminino
Brasil	696	418	761	446	65	28	104	59
Norte	706	432	884	521	178	89	171	93
Nordeste	731	423	831	465	100	42	108	62
Sudeste	678	416	729	441	51	25	101	59
Sul	670	406	675	398	5	-8	70	40
Centro-oeste	686	423	775	463	89	40	124	75
	0-59 anos	60 anos ou mais	0-59 anos	60 anos ou mais	0-59 anos	60 anos ou mais	0-59 anos	60 anos ou mais
Brasil	225	3.199	236	3.514	11	315	23	536
Norte	237	3.286	258	4.316	21	1.030	34	926
Nordeste	249	3.182	264	3.662	15	480	24	560
Sudeste	213	3.196	224	3.446	11	250	23	523
Sul	206	3.191	203	3.185	-3	-6	15	368
Centro-oeste	221	3.256	234	3.727	13	471	28	674

Nota: as taxas são expressas por 100 mil homens ou mulheres e 100 mil habitantes entre 0-59 anos ou com 60 ou mais anos. Padronização das taxas pelo método direto utilizando como padrão a estimativa populacional par a população do Brasil em 2010.

Quanto à análise estratificada por faixa etária, a taxa de mortalidade padronizada no grupo de 0 a 59 anos no Brasil no ano de 2020 foi de 236 óbitos por 100 mil habitantes com essa idade, e a taxa por COVID-19 foi de 23 óbitos por 100 mil habitantes nessa faixa etária. Na faixa etária a partir dos 60 anos, a taxa de mortalidade foi 3.514 óbitos por 100 mil habitantes com essa idade, e a taxa por COVID-19 foi de 536 óbitos por 100 mil habitantes nessa faixa de idade. Nota-se que, no grupo de 60 anos ou mais, a taxa de mortalidade geral foi aproximadamente 15 vezes superior à observada no grupo de 0 a 59 anos. A Região Sul apresentou uma diferença entres as taxas de óbitos inferiores ao observado no país para o grupo de 0 a 59 anos (-3 óbitos por 100 mil habitantes entre 0 a 59 anos), e nas regiões Norte, Nordeste e Centro-oeste esse quantitativo foi superior ao registrado no país (315 óbitos por 100 mil habitantes com 60 anos ou mais), para os indivíduos com 60 anos ou mais (Tabela 2).

No ano de 2020, foram registrados 1.557.169 óbitos por todas as causas no Brasil, sendo 110.434 no Norte (7%), 421.176 no Nordeste (26,7%), 712.333 no Sudeste (45,1%), 108.316 no Centro-oeste (6,9%) e 224.910 no Sul (14,3%). O excesso de óbitos estimado pela razão entre os óbitos observados e esperados, para o ano de 2020, em todo o país, foi de 16%. As regiões Norte (1,27), Centro-oeste (1,21) e Nordeste (1,20) apresentaram 20% ou mais de excesso de óbitos (Tabela 3).

**Tabela 3:** Óbitos esperados e observados em 2020, razão entre os óbitos, excesso de óbitos, e óbitos por COVID-19, no Brasil e regiões.

	Óbitos esperados	Óbitos observados	Razão óbitos	Excesso de óbitos	Óbitos por COVID-19
n (IC95%)	n	% (IC95%)	n (IC95%)	n
Brasil	1.358.353 (1.353.995; 1.362.711)	1.577.169	1,16 (1.16; 1,17)	218.816 (214.458; 223.174)	214.621
Norte	87.055 (86.601; 87.509)	110.434	1,27 (1,26; 1,28)	23.379 (22.925; 23.833)	19.780
Nordeste	350.107 (348.434; 351.780)	421.176	1,20 (1,20; 1,21)	71.069 (69.396; 72.742)	55.029
Sudeste	620.649 (617.829; 623.469)	712.333	1,15 (1,14; 1,15)	91.684 (88.864; 94.504)	98.713
Sul	207.924 (206.665; 209183)	224.910	1,08 (1,08; 1,09)	16.986 (15.727; 18.245)	23.641
Centro-oeste	89.722 (89.260; 90.184)	108.316	1,21 (1,20; 1,21)	18.594 (18.594; 19.056)	17.458

IC95%: intervalo de 95% de confiança.

Nota: a razão foi calculada considerando a divisão dos óbitos observados pelos óbitos esperados, estimados a partir dos óbitos ocorridos nos anos de 2015 a 2019.

A partir da avaliação da mortalidade pelo modelo quasi-Poisson, nota-se um excesso de 218.816 óbitos para o ano de 2020 e um número absoluto de óbitos registrados por COVID-19 (214.621 óbitos) inferior ao excesso de óbitos estimado, nesse ano. Isso também foi observado nas regiões Norte, Nordeste e Centro-oeste. Já nas regiões Sudeste e Sul, o número de óbitos classificados como COVID-19 na causa básica superou o excesso de óbitos por todas as causas (Tabela 3).

Ao avaliar esses parâmetros segundo o sexo, nota-se que a frequência absoluta dos óbitos esperados e dos óbitos observados foi superior no sexo masculino quando comparada ao sexo feminino, para o país e suas regiões. No Brasil, as razões entre os óbitos observados e esperados foram 1,17 e 1,16 nos sexos masculino (17%) e feminino (16%), respectivamente. A Região Norte permaneceu com a maior razão entre as regiões avaliadas em ambos os sexos (1,27), e a Região Sul apresentou a menor razão. Para todas as localidades avaliadas, as razões observadas nos sexos masculino e feminino apresentaram valores próximos (Tabela 4). Os óbitos por COVID-19 foram superiores aos óbitos totais em excesso nas regiões Sudeste e Sul, para os sexos masculino e feminino.

**Tabela 4:** Óbitos esperados e observados em 2020, razão entre os óbitos, excesso de óbitos e óbitos por COVID-19, no Brasil e regiões, segundo sexo e faixa etária.

	Óbitos esperados		Óbitos observados		Razão óbitos (IC95%)		Excesso de óbitos (IC95%)		Óbitos por COVID-19	
	n (IC95%)		n		% (IC95%)		n (IC95%)		n	
	Masculino	Feminino	Masculino	Feminino	Masculino	Feminino	Masculino	Feminino	Masculino	Feminino
Brasil	757.412 (755.532; 759.292)	598.255 (596.747; 599.763)	885.596	690.938	1,17 (1,17; 1,17)	1,16 (1,15; 1,16)	128.184 (126.304; 130.064)	92.683 (75.426; 78.338)	122.689	91.918
Norte	53.365 (53.124; 53.606)	33.481 (33.334; 33.628)	67.709	42.635	1,27 (1,26; 1,27)	1,27 (1,27; 1,28)	14.344 (14.103; 14.585)	9.154 (9.007; 9.301)	12.342	7.436
Nordeste	198.750 (198.041; 199.459)	150.728 (150.164; 151.292)	240.078	180.921	1,21 (1,20; 1,21)	1,20 (1,20; 1,21)	41.328 (40.619; 42.037)	30.193 (29.629; 30.757)	31.031	23.994
Sudeste	336.457 (335.315; 337.599)	284.021 (283.033; 285.009)	388.364	323.682	1,15 (1,15; 1,16)	1,14 (1,14; 1,14)	51.907 (50.765; 53.049)	39.661 (38.673; 40.649)	55.463	43.242
Sul	114.662 (114.133; 115.191)	92.697 (92.287; 93.107)	125.355	99.514	1,09 (1,09; 1,10)	1,07 (1,07; 1,08)	10.693 (10.164; 11.222)	6.817 (6.407; 7.227)	13.569	10.072
Centro-oeste	53.124 (52.891; 53.357)	36.565 (36.399; 36.731)	64.090	44.186	1,21 (1,20; 1,21)	1,21 (1,20; 1,21)	10.966 (10.733; 11.199)	7.621 (7.455; 7.787)	10.284	7.174
	0-59 anos	60 anos ou mais	0-59 anos	60 anos ou mais	0-59 anos	60 anos ou mais	0-59 anos	60 anos ou mais	0-59 anos	60 anos ou mais
Brasil	447.869 (445.927; 449.811)	911.530 (907.874; 915.186)	477.344	1.097.391	1,07 (1,06; 1,07)	1,20 (1,20; 1,21)	29.475 (27.533; 31.417)	185.861 (182.205; 189.517)	49.338	165.258
Norte	39.451 (39.241; 39.661)	47.391 (47.133; 47.649)	42.962	67.175	1,09 (1,08; 1,10)	1,42 (1,41; 1,43)	3.511 (2231; 2663)	19.784 (19.526; 20.042)	5.302	14.475
Nordeste	126.115 (125.519; 126.711)	223.300 (222.257; 224.343)	139.921	280.656	1,11 (1,10; 1,12)	1,26 (1,25; 1,26)	13.806 (14.402; 11.251)	57.356 (56.313; 58.399)	12.837	42.178
Sudeste	185.105 (184.177; 186.033)	434.382 (432.315; 436.449)	196.428	514.636	1,06 (1,06; 1,07)	1,19 (1,18; 1,19)	11.323 (10.395; 12.251)	80.254 (78.187; 82.321)	21.807	76.898
Sul	60.812 (60.404; 61.220)	147.351 (146.400; 148.302)	60.699	164.069	0,99 (0,99; 1,00)	1,11 (1,11; 1,12)	-113 (-521; 295)	16.718 (15.767; 17.669)	4.802	18.839
Centro-oeste	34.312 (34.114; 34.510)	55.335 (55.019; 55.651)	37.334	70.855	1,09 (1,08; 1,09)	1,28 (1,27; 1,29)	3.022 (2.824; 3.220)	15.520 (15.204; 15.836)	4.590	12.868

IC95%: intervalo de 95% de confiança.

Nota: a razão foi calculada considerando a divisão dos óbitos observados pelos óbitos esperados, estimados a partir dos óbitos ocorridos nos anos de 2015 a 2019.

A Tabela 4 mostra que o grupo de indivíduos com idade de 60 anos ou mais apresentou mais que o dobro dos óbitos contabilizados no grupo com idade de 0 a 59 anos no Brasil em 2020. Ainda, os óbitos observados no grupo com 60 anos ou mais foram superiores aos do grupo de 0 a 59 anos para todas as regiões estudadas. O excesso de óbitos foi de 7% para indivíduos entre 0 e 59 anos, e de 20% para aqueles com 60 anos ou mais no país. A razão entre óbitos observados e esperados foi superior à nacional para o grupo de 0 a 59 anos (1,07) nas regiões Norte (1,09), Nordeste (1,11) e Centro-oeste (1,09); e no grupo com 60 anos ou mais (1,20) também nas regiões Norte (1,42), Nordeste (1,26) e Centro-oeste (1,28). Na Região Sul, para o grupo de 0 a 59 anos, não foi observado excesso de óbitos no período (0,99). Os óbitos por COVID-19 foram superiores aos óbitos totais em excesso para ambas as faixas etárias estudadas na Região Sul do país.

## Discussão

Este estudo apontou um excesso de óbitos por todas as causas para a maioria das regiões do país expresso pelos dois métodos empregados, sendo mais acentuado nos subgrupos demográficos do sexo masculino e com 60 anos ou mais de idade. A exceção é a Região Sul, que não apresentou excesso de óbitos geral, no sexo feminino e para ambas as faixas etárias, apresentando diferenças negativas entre as taxas de mortalidade observadas e esperadas em 2020. O uso de taxas de mortalidade padronizadas por idade possibilitou comparações entre subgrupos populacionais, controlando a influência das diferentes composições etárias, especialmente entre as regiões do Brasil e segundo sexo.

A partir do contexto vivenciado, este estudo comparou o número de mortes durante a pandemia de COVID-19 com as mortes esperadas caso a pandemia não tivesse ocorrido. Como vantagem do indicador de excesso de mortalidade, destaca-se a capacidade de aferir mais abrangentemente o impacto total da pandemia, em vez de contar somente as mortes confirmadas pela COVID-19, uma vez que são incluídas na análise causas de mortes mal definidas e mortes por outras causas relacionadas às condições gerais de crise ^17^.

Globalmente, estudos que avaliaram o excesso de óbitos até agosto de 2020 mostram que países como Brasil, França, Itália, Espanha, Suécia, Reino Unido e Estados Unidos apresentaram excesso de mortalidade geral, enquanto Austrália, Dinamarca e Geórgia tiveram redução na mortalidade por todas as causas. A localização geográfica e a sazonalidade de cada país, bem como a diversidade no manejo das medidas de assistência e controle, podem explicar as diferenças observadas nos padrões de mortalidade ^13^.

No Brasil, também foi possível constatar variações entre as grandes regiões do país, observadas na avaliação do excesso de mortalidade geral e estratificado por sexo e faixa etária, independentemente do método utilizado. Os achados confirmam as iniquidades existentes entre essas regiões, como as descritas no relatório do IBGE sobre indicadores sociais ^18^, o qual enfatiza que, embora o acesso a serviços de saúde esteja disponível para toda a população no Brasil, ainda persistem as coberturas diferenciadas que refletem o desenvolvimento heterogêneo do sistema de saúde no tempo e no espaço. O relatório mostra que a restrição de acesso a serviços de saúde esteve mais concentrada na Região Norte (28,3% da população com restrição), e que, enquanto a Região Sul possuía 2,78 leitos por mil habitantes, a Região Norte possuía 2,01. Além disso, em 2020, a razão entre o número de médicos por mil habitantes no país foi de 1,99, variando entre as regiões: Norte (1,07), Nordeste (1,36), Centro-oeste (2,08), Sul (2,32) e Sudeste (2,47) ^18^. As desigualdades sociais no acesso e na utilização dos serviços de saúde relacionam-se com as condições de saúde dos indivíduos, haja vista que grupos mais vulneráveis apresentam maiores taxas de morbidade e mortalidade ^19^. Nesse processo, a disparidade econômica gera um impacto negativo tanto pelo desequilíbrio de oportunidades quanto por limitar a capacidade de resposta diante do contexto de pandemia ^20^.

O Estado do Amazonas, localizado na Região Norte, vivenciou, durante a pandemia da COVID-19, o agravamento da crise sanitária, causado principalmente pelas condições de pobreza e desigualdade social reconhecidas de longa data ^21^, e reforçado pela restrição de acesso e mobilidade dos povos indígenas vivendo nessa região, tornando-os ainda mais suscetíveis à disseminação das formas mais graves da doença ^22^. Na capital do estado, ocorreu um aumento súbito das mortes por COVID-19 no ano de 2020, potencializado pelo desgaste e pela sobrecarga do sistema hospitalar e como resultado das ações governamentais insuficientes voltadas ao enfrentamento das iniquidades sociais, intensificadas principalmente pela pandemia ^21^ ^,^ ^23^.

Em contrapartida, a Região Sul destacou-se pelo menor impacto da pandemia na mortalidade, inclusive não sendo observado excesso nos óbitos em alguns estratos populacionais nessa região do país. Historicamente, a carga de doenças nas regiões Sul e Sudeste é inferior àquela observada nas regiões Norte e Nordeste, condição que também se refletiu na pandemia de COVID-19 ^24^ ^,^ ^25^. Além disso, a expectativa de vida saudável ^26^ e o desenvolvimento humano são superiores nas regiões Sudeste e Sul do Brasil ^27^ ^,^ ^28^. Um estudo mostrou que o excesso de mortes avaliado nos primeiros seis meses de pandemia apresentou heterogeneidade quanto à localização geográfica, sendo observada mortalidade em excesso em momentos mais precoces na Região Norte, em comparação ao Sudeste e ao Sul. Os autores reforçam a existência de diferentes gradientes de mortalidade por COVID-19 entre as regiões ^26^.

Uma análise descritiva inicial sobre o excesso de mortalidade observado nos meses de março a maio de 2020 indicou excesso de 39.146 óbitos para o período estudado, sendo maior entre homens em comparação com as mulheres. Esse aumento foi maior nas capitais das regiões Norte, Nordeste e Sudeste ^29^, corroborando os dados apontados neste estudo.

Um excesso de mortalidade também foi observado em 23 países europeus ^30^, que apresentaram uma taxa de mortalidade significativamente maior em homens quando comparado com as mulheres (razão de risco = 1,60). Considerando os aspectos sociais de gênero, esse resultado pode estar relacionado aos hábitos masculinos, já que eles procuram menos pelos serviços de saúde, fumam mais e higienizam as mãos com menor frequência do que mulheres ^31^ ^,^ ^32^.

Um estudo que estimou o excesso de mortes nas cidades de Manaus (Amazonas), Fortaleza (Ceará), Rio de Janeiro e São Paulo, de fevereiro a junho de 2020, registrou 74.410 mortes por causas naturais nas quatro cidades, com excesso de 46% em relação ao esperado para o período. O maior excesso de mortes ocorreu em Manaus (112%), seguido por Fortaleza (72%), Rio de Janeiro (42%) e São Paulo (34%). O excesso de mortes também foi maior nos homens ^31^, apoiando os resultados deste estudo. Ainda, a investigação do excesso de óbitos por causas específicas no primeiro semestre de 2020 no Município de São Paulo mostrou que a COVID-19 foi responsável por 94,4% do excesso de mortes no município e que a mortalidade pela doença foi duas vezes maior para homens do que para mulheres ^33^, também corroborando os achados deste estudo.

Para todos os estados do Brasil no ano de 2020, dados apontaram um excesso de óbitos de 22,2%, sendo maior para os homens (25,2% nos homens e 19% nas mulheres) e negros (27,8% em negros e 17,6% em brancos) ^34^. Embora não se tenha avaliado, por ora, o excesso de mortalidade considerando a raça/cor da pele, esses resultados reforçam a importância e a necessidade de análises complementares, que considerem as desigualdades raciais, observadas historicamente no país.

No que se refere aos grupos etários, outro estudo também apontou maior excesso de óbitos entre os indivíduos com maior idade. Um conjunto de 24 países/estados federativos europeus, integrantes da Rede Europeia de Monitoramento do Excesso de Mortalidade para Ações de Saúde Pública (*European Monitoring of Excess Mortality for Public Health Action* - EuroMOMO), relataram que, no período de março a abril de 2020, o excesso de mortalidade afetou particularmente indivíduos com idade igual ou superior a 65 anos de idade (91% de todas as mortes em excesso) ^35^.

Nas regiões Norte, Nordeste e Centro-oeste, o excesso geral de óbitos foi superior ao número de óbitos por COVID-19, indicando um impacto indireto da pandemia. Um excesso de mortes não ocasionadas diretamente pela doença e um alto número de mortes em domicílios e/ou vias públicas também foram identificados em Manaus, o que pode indicar uma subnotificação das mortes por COVID-19 e a necessidade da revisão das causas de mortes pela vigilância epidemiológica, principalmente aquelas associadas a sintomas respiratórios ^31^.

De acordo com pesquisadores da Universidade Johns Hopkins, nos Estados Unidos, que avaliaram o excesso de mortalidade em vários países na pandemia, alguns fatores podem influenciar na relação entre o excesso de mortes geral e o número de mortes por COVID-19, e esses números podem diferir por vários motivos. Entre eles, o fato de que alguns países registram apenas as mortes por COVID-19 ocorridas em hospitais, havendo subnotificação referente às pessoas que morrem em residências ou outros espaços extra-hospitalares; ou ainda relatam apenas mortes com confirmação laboratorial da COVID-19, excluindo indivíduos não testados ^17^. Essa pode ser uma possível explicação para as menores magnitudes observadas na Região Sul do país. A taxa de notificação de casos de COVID-19 no Brasil foi estimada em 9,2% em um estudo publicado em 2020, dificultando o conhecimento real da dimensão da pandemia e consequentemente a tomada de decisões ^36^. Vale ressaltar que a negação da doença, vivenciada especialmente no primeiro ano da pandemia, refletiu no registro dos dados da COVID-19 no país, dificultando o acesso à informação em saúde pela população em um momento oportuno para a adoção de ações de controle efetivas. Em suma, os sistemas de notificação de óbitos podem ser insuficientes para mensurar com precisão a mortalidade, principalmente em países com menos recursos humanos e financeiros ^17^.

Além disso, a pandemia pode resultar no aumento de mortes por outras causas, mal definidas inclusive, por diversas razões, como: o enfraquecimento do sistema de saúde; a dificuldade na adesão ao acompanhamento de outros agravos, motivados pelo isolamento social ou barreiras de acesso; e a redução do financiamento destinado ao tratamento de outras doenças, como HIV/aids, malária e tuberculose ^37^, gerando impacto potencial ainda mais desastroso a longo prazo ^38^. Por outro lado, a pandemia também pode contribuir para a diminuição de mortes por outras causas, por exemplo as ocasionadas por causas externas, como acidentes rodoviários, devido às restrições de mobilidade ^17^.

Em síntese, as mortes confirmadas por COVID-19 e o excesso de mortalidade podem estar relacionadas de maneiras que não são diretas, já que esses indicadores abordam perspectivas distintas, com características particulares. Enquanto as mortes confirmadas por COVID-19 podem subestimar o impacto da pandemia no total de mortes em comparação com o excesso de mortalidade geral, este último, por sua vez, não apresenta informações sobre as causas das mortes. Apesar de tais indicadores apresentarem vantagens e desvantagens, ambos são, em conjunto, essenciais para entender o impacto da pandemia na mortalidade de forma ampla ^17^.

Algumas limitações do estudo devem ser mencionadas. Apesar de este estudo não ter avaliado especificamente os óbitos por causa mal definidas, entende-se que os indicadores de COVID-19 possam estar subestimados, modificando a interpretação da magnitude mínima da mortalidade pela doença, que estaria possivelmente mais elevada em função do sub-registro de óbitos em decorrência, especialmente, das causas mal definidas. Ainda, o cálculo do indicador do excesso de mortalidade de forma geral não permitiu identificar quais causas contribuíram diretamente para o excesso de mortalidade, o que possibilitaria dimensionar e diferenciar o impacto direto e indireto da pandemia de COVID-19. As análises por causas têm mostrado que, além do excesso de óbitos já esperado por doenças infecciosas e parasitárias ^5^, houve um excesso de mortes maternas ^39^, bem como uma redução da mortalidade atribuível a doenças do coração, neoplasias malignas ^40^, doenças respiratórias e causas externas ^37^. Portanto, para compreender melhor o cenário da pandemia no Brasil, propõe-se a realização de análises que considerem os grupos de causas de mortes, além do efeito na gestão dos serviços de saúde. Destaca-se a importância de que essas análises considerem o sexo, as faixas etárias e a raça/cor da pele, com o objetivo de identificar grupos mais vulneráveis.

Tendo em vista o exposto, conclui-se que os dados trabalhados permitiram um entendimento mais abrangente do impacto da pandemia na mortalidade do Brasil em 2020, verificando por meio de métodos distintos que indivíduos do sexo masculino e com 60 anos ou mais foram mais afetados pela mortalidade em excesso, principalmente quando residentes na Região Norte do país. Ressalta-se a consolidação do indicador de mortalidade geral padronizada como uma ferramenta válida para estimar o excesso de mortes no contexto da pandemia, por ser um parâmetro robusto e que possibilita a comparação espacial entre as localidades; enquanto a mensuração da mortalidade específica por COVID-19 é um indicador que depende da organização e do financiamento dos serviços de saúde para a disponibilização de diagnósticos laboratoriais, sendo esse ponto um dificultador no cenário brasileiro, com escassez de testes, especialmente no primeiro ano da pandemia e em regiões mais remotas. Entretanto, conhecer ambos os indicadores, considerando as particularidades de cada grupo, pode auxiliar gestores e profissionais de saúde na compreensão sobre os dados causados pela pandemia, bem como no manejo das ações de prevenção de novos casos.
